# Treatments in Alzheimer’s disease

**DOI:** 10.1007/s00415-017-8395-1

**Published:** 2017-01-24

**Authors:** G. A. Malik, N. P. Robertson

**Affiliations:** Institute of Psychological Medicine and Clinical Neurosciences, Cardiff University, Cardiff, CF14 4XW UK

## Introduction

Alzheimer’s disease (AD) is the commonest cause of dementia worldwide. However, no treatment is yet available to halt or reverse the underlying pathology of established disease. Advances in the management of other common diseases and an improvement in general health have resulted in an increasingly elderly population so that the prevalence of AD is expected to increase significantly over time. In 2015, almost 47 million people worldwide were estimated to be affected by dementia, with estimates of 75 million by 2030, and 131 million by 2050, with the greatest increase expected in low-income and middle-income countries. The socio-economic burden of dementia has become immense and the development of effective interventions has become one of the greatest priorities for health care research in the twenty-first century.

Current treatments for AD include acetyl cholinesterase inhibitors and the N-methyl-D-aspartate receptor antagonist Memantine, but offer only symptomatic rather than disease-modifying benefits. In spite of the urgent requirement, disease-modifying therapies have so far proved elusive, in particular with almost universally disappointing results in late stage clinical trials for therapies that target aspects of amyloid metabolism. However, results from a recent phase 1b trial, published in 2016, have offered new hope. Aducanumab, a human monoclonal antibody that selectively targets aggregated Aβ, has been shown to remove the build-up of the Alzheimer’s protein amyloid in the brain and slow the decline in memory and thinking skills in people with AD. This month’s journal club looks at this and two other papers reporting recent clinical trials in AD which together offer a window into potential future therapeutic directions in this devastating disorder.

## The antibody aducanumab reduces Aβ plaques in Alzheimer’s disease

This paper provides interim results from a double-blind, placebo- controlled phase 1b randomized trial (PRIME). Its primary outcome was to investigate safety, tolerability, pharmacokinetics, and pharmacodynamics of monthly infusions of aducanumab in patients with prodromal or mild AD with brain Aβ pathology confirmed by molecular positron emission tomography (PET) imaging. 165 participants were randomized across 33 centres to receive monthly infusions of placebo, or 1, 3, 6, or 10 mg/kg doses of aducanumab for 12 months. 40 patients dropped out, leaving between 21 and 32 individuals per group for analysis. The primary outcome was reduction in brain Aβ plaques as measured by florbetapir PET imaging in a dose- and time-dependent fashion at baseline, six months, and 12 months. Additional cognitive tests were also performed but were exploratory as the study was not powered to detect clinical change.

At 6 and 12-month intervals there was a significant dose-dependent reduction of Aβ deposits. In the placebo group, the florbetapir standardized average uptake value ratio (SUVR) was 1.44, compared to 1.16 in the 10 mg/kg group. The reduction in plaque load was seen in participants with both prodromal and mild AD, and regardless of ApoE4 carrier status and reduced brain Aβ in a dose- and time-dependent manner. The most common adverse effects were amyloid-related imaging abnormalities (ARIA), headache, urinary tract infection and upper respiratory tract infection. ARIA-vasogenic oedema occurred in no patients in the placebo group but up to 41% of the high dose group. ARIA although not fully understood, generally occurred early in the course of treatment and is likely to be related to increased extracellular fluid, however, no patient were hospitalised for this and there were no drug related deaths.

*Comment and conclusion*. Based on its primary outcomes the PRIME study shows that aducanumab penetrates the brain and decreases Aβ in patients with AD in a time and dose-dependent manner. With only 165 participants the study was not powered to detect clinical change so that the preliminary findings of a slowing of clinical decline measured by Clinical Dementia Rating (Sum of Boxes and Mini Mental State Examination scores versus placebo at 12 months) must be interpreted with some caution. The greatest effect in reducing brain Aβ plaques was observed in the 10 mg/kg group (*p* < 0.05 versus placebo), and whereas significant Aβ reduction was detectable by 6 months, any clinical effects were not seen until 12 months. Analysis also suggested that performance on the CDR-SB and the MMSE stabilized only in patients who had a substantial reduction in amyloid at one year. Patients for whom there was no reduction in imaging correlates of Aβ levels, declined cognitively in a similar pattern to the placebo group.

The authors acknowledged several study limitations of the PRIME phase 1b study, including staggered parallel-group design, small sample sizes, limited region (USA only), and possible partial unblinding due to the imaging frequency required following ARIA. However, this study suggests that a reduction of brain Aβ may confer a clinical benefit, thereby supporting the amyloid hypothesis and providing impetus to research in this field, including unravelling the exact molecular basis of clearance of Aβ clearance from the brain following aducanumab. The results from this Phase 1b study support results from earlier preclinical trials demonstrating aducanumab brain penetration, target engagement, and dose-dependent clearance of Aβ plaques, and if confirmed in future studies powered to detect clinical benefit studies would provide support for aducanumab as an Aβ-removing, disease-modifying therapy for AD.

Sevigny J et al (2016) Nature 537:50–56.

## Efficacy and safety of tau-aggregation inhibitor therapy in patients with mild or moderate Alzheimer’s disease: a randomized, controlled, double-blind, parallel-arm, phase 3 trial

Based on the previous potential efficacy of methylthioninium chloride in patients with AD, a modified stable reduced form of the methylthioninium moiety was identified as a potential therapeutic agent. Leuco-methylthioninium bishydromethanesulfonate (LMTM) acts as a selective inhibitor of tau protein aggregation both in vitro and in transgenic mouse models. The aim of this study was to determine whether LMTM was safe and effective in modifying disease progression in patients with mild to moderate AD. The primary outcome measure was progression on the Alzheimer’s Disease Assessment Scale-Cognitive Subscale (ADAS-Cog) and the AD Co-operative Study-Activities of Daily Living Inventory (ADCS-ADL) scales from baseline, assessed at week 65 in the modified intention-to-treat population.

This was a large, multinational trial of 15 months duration. It was a randomized, controlled, double-blind, parallel-group trial involving 115 academic centres and private research clinics in 16 countries in Europe, North America, Asia, and Russia involving patients younger than 90 years with mild to moderate Alzheimer’s disease. Patients taking other medicines for Alzheimer’s disease were not excluded as the study group felt it infeasible not to allow their inclusion. Participants were randomly assigned (3:3:4) oral tablets to 75 mg LMTM twice a day, 125 mg LMTM twice a day, or control (4 mg LMTM twice a day to maintain blinding with respect to urine or faecal discolouration). Randomisation was achieved via an interactive web response system and all participants and all assessors were masked to treatment assignment throughout the study.

In total 891 participants were assigned to treatment (357 to control, 268–75 mg LMTM twice a day, and 266–125 mg LMTM twice a day). Neither co-primary outcomes demonstrated treatment benefit at any dose: ADAS-Cog score compared with control (*n* = 354, 6.32, 95% CI 5.31–7.34): 75 mg LMTM twice a day (*n* = 257) −0.02, −1.60 to 1.56, *p* = 0.9834, 125 mg LMTM twice a day (*n* = 250) −0.43, −2.06 to 1.20, *p* = 0.9323 and change in ADCS-ADL score compared with control (−8.22, 95% CI −9.63 to −6.82): 75 mg LMTM twice a day −0.93, −3.12 to 1.26, *p* = 0.8659; 125 mg LMTM twice a day −0.34, −2.61 to 1.93, *p* = 0.9479). The most common adverse events were gastrointestinal and urinary effects with both high doses of LMTM, and were the commonest causes for discontinuation. Non-clinically significant dose-dependent reductions in haemoglobin concentrations were the most commonly identified laboratory abnormality. Amyloid-related imaging abnormalities were noted in less than 1% (8/885) of participants.

*Comment and conclusion.* As with previous study’s targeting tau protein accumulation analysis of the primary outcomes for this study was negative and the results do not suggest benefit of LMTM as an add-on treatment for patients with mild to moderate Alzheimer’s disease. Findings from a recently completed 18-month trial of patients with mild Alzheimer’s disease are to be released in the near future.

Gauthier S et al (2016) The Lancet 388(10062):2873–2884.

## Towards personalised intervention in Alzheimer’s disease

This final paper by Peng et al. is a review of the current understanding of risk factors and intervention strategies in AD and provides a practical alternative approach in addressing the considerable challenges of treating patients with Alzheimer’s. The authors suggest that these challenges commonly occur as a result of the heterogeneity in disease aetiology, which involves complex and divergent pathways and that more detailed patient stratification is required to inform intervention strategies which can be tailored for individual patients. They argue that this approach would provide improved patient management more focussed clinical trials and would also have potential for more personalised intervention in AD.

The authors suggest that the required information that would allow improved patient stratification centres on demographic and biological data. The demographic data includes *APOE* genotype, age, gender, education, environmental exposure, life style, and medical history, whereas the biological data might include genome sequencing, imaging, biomarkers and functional assays on patient-specific induced pluripotent cells. Intervention strategies based on physical activity, brain stimulation, social communication, diet and drugs are reviewed as well as a stage specific intervention strategy.

*Comment and conclusion.* Admittedly intervention strategies are limited currently and this review paper offers a practical approach to modify risk factors and better target preventative treatments. It is proposed that the demographic and in-depth information can also generate focussed preventive strategies to limit the rate of conversion from mild cognitive impairment to dementia.

Peng X et al (2016) Genomics Proteomics Bioinformatics 14:289–297.

